# A Novel Glass Polyalkenoate Cement for Fixation and Stabilisation of the Ribcage, Post Sternotomy Surgery: An *ex-Vivo* Study

**DOI:** 10.3390/jfb4040329

**Published:** 2013-11-21

**Authors:** Adel M.F. Alhalawani, Declan J. Curran, Belinda Pingguan-Murphy, Daniel Boyd, Mark R. Towler

**Affiliations:** 1 Department of Biomedical Engineering, Faculty of Engineering, University of Malaya, Kuala Lumpur 50603, Malaysia; E-Mails: adel.alhalawani@ryerson.ca (A.M.F.A.); bpingguan@gmail.com (B.P.-M.); 2 Department of Mechanical & Industrial Engineering, Ryerson University, Toronto M5B 2K3, ON, Canada; E-Mail: curran@ryerson.ca; 3 Department of Applied Oral Sciences, Faculty of Dentistry, Dalhousie University, Halifax B3H 4R2, NS, Canada; E-Mail: d.boyd@dal.ca

**Keywords:** sternal fixation, gallium, glass polyalkenoate cement, sternotomy, compressive strength, biaxial flexural strength, roughness, contact angle, bovine sterna

## Abstract

This study investigates the use of gallium (Ga) based glass polyalkenoate cements (GPCs) as a possible alternative adhesive in sternal fixation, post sternotomy surgery. The glass series consists of a Control (CaO–ZnO–SiO2), and LGa-1 and LGa-2 which contain Ga at the expense of zinc (Zn) in 0.08 mol% increments. The additions of Ga resulted in increased working time (75 s to 137 s) and setting time (113 to 254 s). Fourier Transform Infrared (FTIR) analysis indicated that this was a direct result of increased unreacted poly(acrylic acid) (PAA) and the reduction of crosslink formation during cement maturation. LGa samples (0.16 wt % Ga) resulted in an altered ion release profile, particularly for 30 days analysis, with maximum Ca^2+^, Zn^2+^, Si^4+^ and Ga^3+^ ions released into the distilled water. The additions of Ga resulted in increased roughness and decreased contact angles during cement maturation. The presence of Ga has a positive effect on the compressive strength of the samples with strengths increasing over 10 MPa at 7 days analysis compared to the 1 day results. The additions of Ga had relatively no effect on the flexural strength. Tensile testing of bovine sterna proved that the LGa samples (0.16 wt % Ga) are comparable to the Control samples.

## 1. Introduction

Sternotomy and sternal closure occur before and after cardiac surgery, respectively. Sternotomy is utilized during thoracic procedures such as open-heart, cardiac valve replacement and coronary bypass surgeries [1,2]. Even compared to less-invasive surgeries such as lateral thoracotomy, it remains the preferred choice for surgeons to reach the thoracic cavity, due to its lower levels of associated pain and incidence of respiratory complications [3]. However, post-operative complications such as instability, deep sternal wound infection (DSWI) and non-union are associated with poor sternal fixation resulting in mortality, morbidity and resource utilization [4,5,6,7,8,9,10,11,12,13]. Alhalawani and Towler [14] conducted a systematic review of sternal closure techniques including wiring, interlocking, plating and cementation; discussing the relative advantages and disadvantages of each. It was concluded that all of these techniques have complications restricting their widespread adoption. Alhalawani and Towler also suggested the ideal characteristics for a sternal closure device in order to contribute to reduced post-operative complications; these characteristics include a device with mechanical properties suited to the local environment, biocompatibility, radio-opacity, cost-effectiveness and ease-of-removal when necessary.

Glass polyalkenoate cements (GPCs) are traditionally used in dental restorative and luting applications [15]. These aqueous cements set by an acid base reaction between the silicate-based glass and the acidic polymer (poly(acrylic acid)—PAA) in the presence of water [16]. Therefore, their chemistry gives them the potential for use in a range of orthopedic applications [17] including vertebroplasty and kyphoplasty. GPCs are both biocompatible and bioactive [18,19,20,21,22,23,24,25,26], but the presence of aluminum (Al^3+^) in the glass phase of GPCs has restricted their use in orthopedics due to both its influence on bone mineralization [27,28] and its associated involvement in the pathogenesis of degenerative brain diseases [21,29,30]. It was reported by Carter *et al.* [31] that the glass composition can be modified by incorporating ions which have a positive therapeutic effect upon release from the cement. Hence, researchers have investigated alternative compositions of the glass phase of GPCs by utilizing elements such as zirconium (Zr), zinc (Zn), silver (Ag), strontium (Sr), germanium (Ge) and titanium (Ti), often for the purposes of replacing Al^3+^ [18,19,20,21,23,26]. Zr has been reported to extend the setting reaction of GPCs formulated from a glass phase containing it; an extended setting reaction of 5–10 min is usually required by the surgeon [32]. In addition, Zn has a number of positive effects on the body, for example; the ability to increase the deoxyribonucleic acid (DNA) of osteoblasts [33], resulting in increased bone mass [34], and antibacterial effects [35].

The importance of gallium (Ga^3+^) ions and its compounds in the medical field are widely reported in the literature [24,36,37,38,39,40]. Its chemotherapeutic potential was discovered in 1971 [40], and the Ga ion has since been reported to have anti-bacterial [37,39,40,41,42,43], anti-inflammatory [40] and anti-cancerous effects [39]. Its anti-bacterial effect is efficient against both fungi [44] and pathogenic bacteria [45,46,47] while Ga compounds demonstrated anti-tumor activity in the treatment of non-Hodgkin lymphoma, colorectal cancer and carcinoma of the urothelium [48]. Ga is also used as a coating for radiopharmaceuticals in order to obtain molecular tumor imaging due to its short half-life of approximately 68 min [40]. Ga increases bone calcium levels [34] by inhibiting resorption, which is effective in controlling both cancer-related hypocalcaemia and bone resorption [49]. Hence, Ga-containing GPCs have the potential to elicit a range of positive responses from surrounding tissue when utilised in orthopedic applications [24]. Depending on the concentration, Ga can act as both a glass former and modifier when incorporated into the GPC [50], consequently it has been reported [24] that Ga incorporation into Al-free GPCs can help modulate and improve the handling characteristics of these GPCs.

Existing fixation devices for sternotomy fixation and repair have not gained widespread adoption [14]. Techniques including bands, staples, or wires are cost-effective however these fixation methods are unable to provide sufficient stabilisation, especially for patients with additional risks such as osteoporosis. Plating techniques are more successful in reducing post-operative complications when compared to wiring techniques, but wiring is more practical and cost effective. In general, serious post-operative complications were associated with the use of both approaches in high-risk patients. Screw loosening, long-term follow-up and use of an incorrect plate size were the major complications associated with the plating technique, while instability, wire ingress into the bone, and wound infection, post-surgery, were associated with wiring. A small number of studies have analyzed the use of interlocking systems for sternal fixation. Plating and interlocking techniques are superior to wiring in terms of stability and post-operative complications; however, further clinical studies, including long-term follow-up, are required. 

Kryptonite^TM^ cement (a polymeric material synthesised from castor oil and calcium carbonate powder) has been shown to prevent sternal displacements when used in conjunctions with wires cerclage closure due to good adhesive and strength properties [4,51]. In addition, it has been shown to have bioabsorbable and osteoconductive properties [52,53]. However, wires are still needed in the first few hours to allow the kryptonite to set. In addition, a study by Mastrobuoni *et al.* [54] conducted on 10 patients found that Kryptonite did not show any significant benefit on sternal healing, while one patient developed a deep sternal wound infection. Thus although Kryptonite is, at present, the best choice of adhesive material for sternal closure; it still relies on the use of wires in the initial few hours, it does not work to combat the possible wound infection and it has been implicated as not providing any significant benefit to healing.

This initial study evaluates the potential of using a therapeutic, anti-bacterial, Ga-containing GPCs for sternal fixation by characterising a series of Ga-containing GPCs with respect to their applicability for sternotomy fixation and repair without the use of fixation wires. In particular this paper correlates chemistry underpinning the working time, setting time, compression strength and biaxial flexural strength of novel GPCs with a series of new glass compositions. Furthermore, *ex-vivo* evaluations relating to sternotomy were undertaken. 

## 2. Results and Discussion

This study was undertaken in order to determine the suitability of GPCs synthesised from Ga-containing glasses, for use in fixation and repair of the rib-cage, post sternotomy. X-ray diffraction (XRD) confirmed that all fired glasses were fully amorphous (data not shown). Each glass was then subjected to particle size analysis (PSA) and all glasses were confirmed to have similar mean particle diameters, ranging between 4.7 and 3.9 μm for Control and LGa-2, respectively.

**Figure 1:**
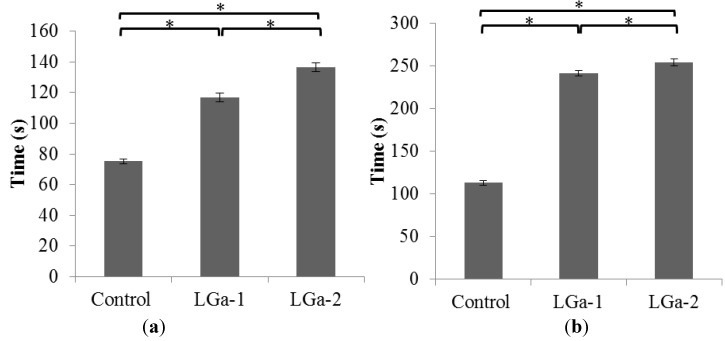
Rheological properties (**a**) Working time; (**b**) Net setting times. Stars and bars show statistical significance (p < 0.05).

The rheological properties of the cements formulated from these glasses were of significant importance. Those properties were assumed to provide some indications relevant to the role of the Ga^3+^ ions in changing the working (T_w_) and net setting (T_s_) times. Figure 1 presents the T_w_ (Figure 1a) and T_s_ (Figure 1b) of the cement series. T_w_ increased, from ~75 to 137 s, as the concentration of Ga increased from 0.00 to 0.16 mol%. Similar behavior was also obtained for T_s_, which increased from ~113 to 254 s. There was a statistically significant difference (P < 0.05) between the three glasses in the series when tested for T_w_ and T_s_. A significant increase in T_w_ and T_s_ was also realized with the addition of Ga; T_w_ increased from ~117 to 137 s for LGa-1 and LGa-2 respectively, while T_s_ increased from ~ 242 to 254 s for LGa-1 and LGa-2 respectively. This indicates that the addition of Ga to the glass phase provides a material with handling properties more suited to sternotomy applications since the T_w_ (~75 s) and T_s_ (~113 s) of the Control glass are considered short. 

**Figure 2:**
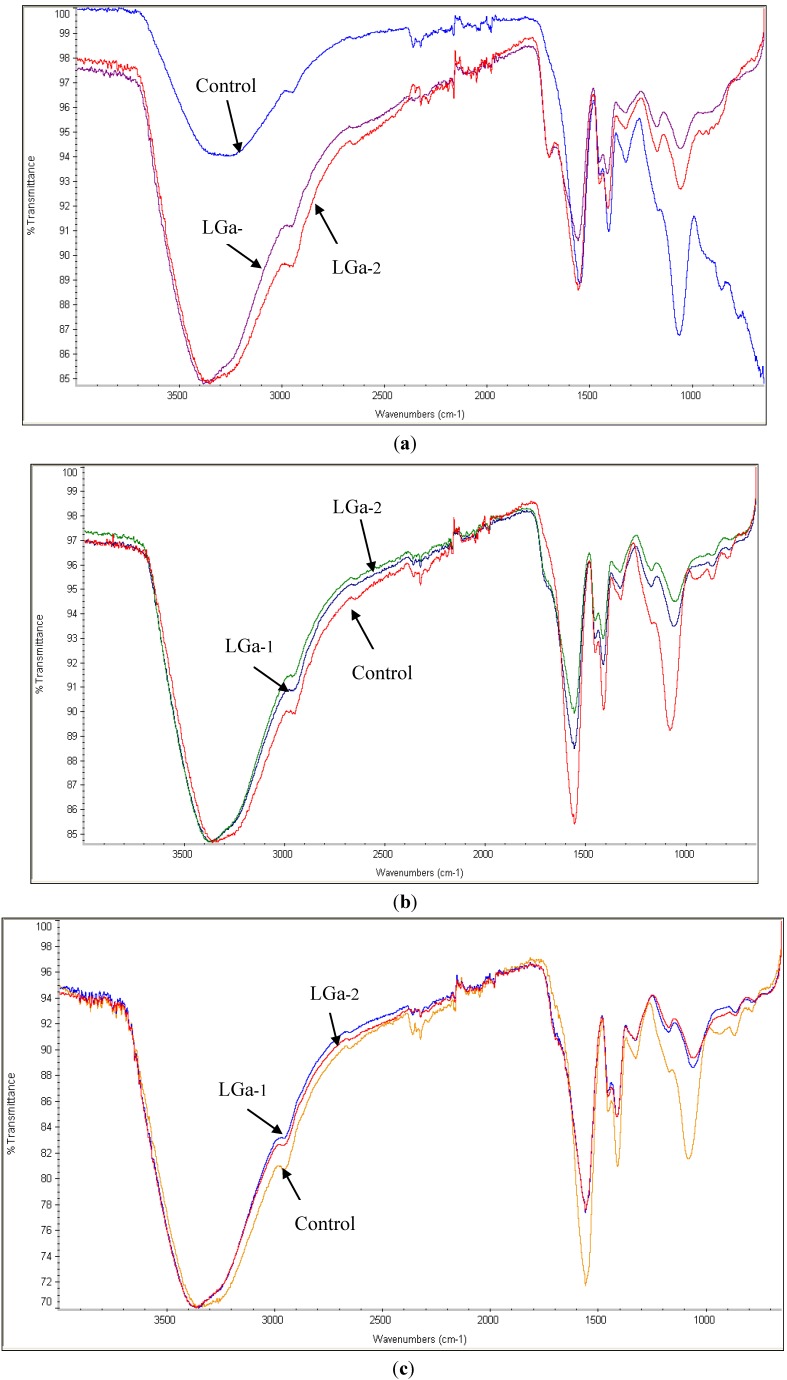
FTIR spectrum of cement series over (**a**) 1 day; (**b**) 7 days; (**c**) 30 days post cement preparation.

Fourier transform infrared (FTIR) spectroscopy was conducted to investigate the reaction kinetics between the glass powder and the PAA. FTIR spectra obtained for the grounded cement powder at 1, 7 and 30 days post cement preparation and storage in distilled water are shown in Figure 2a–c respectively. Similar trends were observed for the Control, LGa-1 and LGa-2 cement series through GPC maturation. The broad peak at 3200 to 3300 cm^−1^ was observed for all spectra (Figure 2), which is assigned to the O-H stretch of adsorbed water [55]. Figure 2a shows that the addition of Ga resulted in a greater water absorption in LGa-1 and LGa-2, ~85% Transmission (%t), when compared with the Control glasses, ~95%t. Figure 2b shows that water absorption increased for the Control (~85%t) with cement maturation over 7 days while it remained constant for LGa-1 and LGa-2 (~85%t). However, an increased intensity (~70%t) of O-H group was observed for 30 day measurements (Figure 2c), implying that all cement formulations increased O–H content when aged. The shoulder peak at ~1700 cm^−1^ (Figure 2a) is assigned to the un-reacted carboxyl (COOH) functional group in the PAA [56]. In the 1 day samples the Control does not display this peak while both LGa 1 and 2 samples do. Thus the presence of Ga in the 1 day samples inhibits COOH reactions resulting in the presence of un-reacted COOH functional groups. The presence of un-reacted COOH in the 1 day LGa samples (Figure 2a) implies that there would also be increased levels of un-reacted COOH in these samples during the mixing and working stages, which would explain the increased wettability of these samples during the working and setting regimes [57,58]*.* This is not the case for the 7 and 30 day LGa samples, which show an increase in transmission in the 1700 cm^−1^ wavenumber. Thus the level of un-reacted COOH diminishes irrespective of Ga content, which may indirectly imply an increase in cross-linking in the cements. Although the level of cross-linking can be more directly measured using the 1550 cm^−1^ and 1400 cm^−1^ peaks. Both Zhang *et al.* [59] and Rajamathi *et al.* [60] assign the 1550 cm^−1^ peak to the asymmetric stretching vibration of the carboxyl COO, which could be assumed to be an asymmetrically bonded COO–X molecule, where X represents a possible metal cation, due to its asymmetric nature. This was shown to be the case by Matsuya *et al.* [61] where they attribute this peak to the asymmetric vibration between the bonded COO– and Ca^2+^ ions. In an in-depth FTIR study by Maltsev and Shevelkov [62] this peak is assigned to the Ga^3+^ ions, which most likely is the Ga content of the COO–Ga (metal carboxylate) molecule. Thus it is safe to assume that this peak highlights the level of cross-linking (bonding) between the dissociated COO– group and metal cations, such as the Ca^2+^, Ga^3+^ and Zn^2+^, to form a metal carboxylate and thusly can serve as an indicator for increased or decreased cross-linking in the cements [56]. The intensity of the ~1550 cm^−1^ peak of the 1 day samples was found to be relatively similar, with the Control and LGa-2 samples having 89%t while the LGa-1 samples show 90%t, therefore there is effectively no difference in the level of cross-linking between these samples at 1 day. At 7 days the LGa samples experience relatively no change in %t, while the Control sample experiences a drop in %t with an increase in the peak height, of 3%, to 86%t. Thus, there is a slight increase in the level of bonded COO– in these samples. At 30 days all samples experience a drop in transmission at this wavenumber with the Control dropping to 72%t and the LGa 1 and 2 samples dropping to 78%t. Therefore aging the samples resulted in an increase in the level of reacted COO–. The peak at ~1400 cm^−1^ is assigned to the symmetric metal carboxylate [63,64] in much the same manner as the ~1550 cm^−1^ peak is for the asymmetric metal carboxylate ion. In the 1 day samples, the Control, LGa-1 and LGa-2 present similar peak intensities with 91, 93 and 92%t. After 7 days the only change experienced by this peak is in the LGa-2 samples with a slight decrease in peak height to 94%t. The real change occurs in the 30 day samples. The Control experiences the largest increase in peak height with an increase to 82%t, while the LGa 1 and 2 samples experience a drop in transmission to 86%t. In a similar manner to the 1550 cm^−1^ peak, as the samples age, the level of symmetric COO and metal ion bonding increases, effectively increasing the level of cross-linking in the cements. The 1070 cm^−1^ peak represents the Si–O–Si bridges [65] of the cements and as such its relative increase or decrease in intensity correlates to an increase or decrease in the formation of bridging oxygens. Over the full 30 days, both LGa 1 and 2 samples experience negligible change in the transmission levels of this peak and remain constant at 94 cm^−1^ and 93 cm^−1^ respectively, implying that the number of Si–O–Si remains constant when aged. The Control samples experience fluctuation over the 30 days, ranging from 87%t, 89%t and 82%t for the 1, 7 and 30 day samples, respectively. Thus there is effectively no change in the %t at this wavenumber until the 30 day samples, implying that the level of Si–O–Si bonds increases when aged in water.

**Figure 3:**
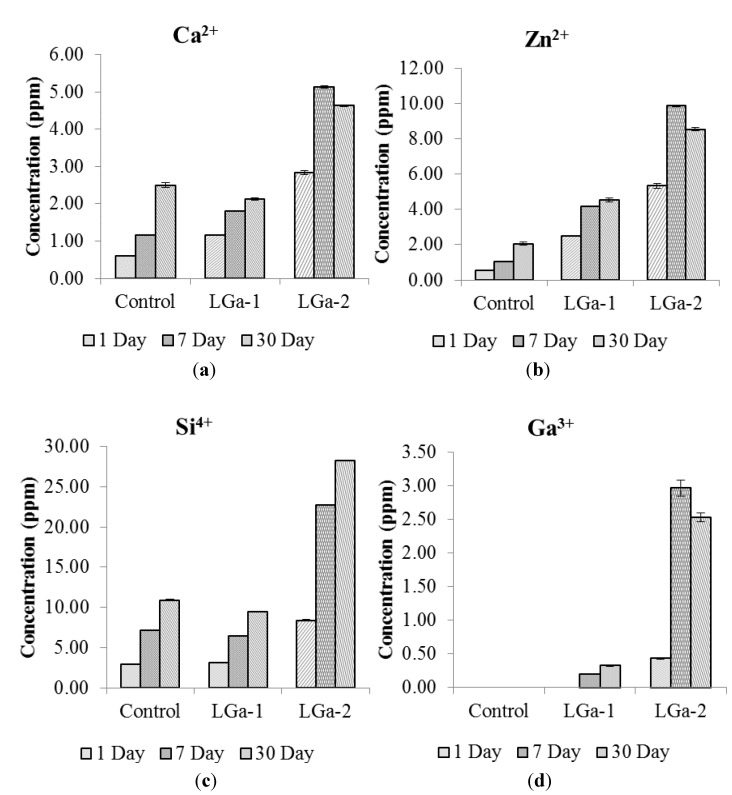
Ion release profiles for cement series over 1, 7 and 30 days (**a**) Ca^2+^; (**b**) Zn^2+^; (**c**) Si^4+^; and (**d**) Ga^3+^.

Ion release studies were performed in order to evaluate the solubility of the cement series in relation to Ga incorporation into the glass phase. These studies are significant to determine the therapeutic effect that these cements might have upon implantation into the skeleton. Figure 3 shows the ion release profiles over 1, 7 and 30 days. Figure 3a shows the release profile of Ca^2+^ over 1, 7 and 30 days post cement preparation. Ca^2+^ release was found to increase for 1 and 7 days for all cement series and for 30 days for Control and LGa-1 cements. The maximum release of Ca^2+^ ions was ~5 part per million (ppm) for LGa-2 at 7 days evaluation. It can be noted that the Ca^2+^ release profile increases with the addition of Ga. However, similar results were obtained for the Control and LGa-1 cements. This is expected due to the low concentration of Ga (0.08 mol%) in the LGa-1 cement in comparison with the 0.16 mol% incorporated for the LGa-2. Figure 3b displays the Zn^2+^ release from the cements. Similar results to Ca^2+^ are obtained, with higher concentrations for Zn^2+^ occurring over 1, 7 and 30 days. Moreover, similar to the Ca^2+^ release profile, the maximum Zn^2+^ release was obtained for LGa-2 at 7 days, which peaked at ~10 ppm. Results of Zn^2+^ release (Figure 3b) show that the concentration of Zn increases with the addition of Ga in the starting glass, however, the mol% of Zn in the starting glasses decreases from 0.40 to 0.24 mol%. This is expected due to the longer setting reaction resulting from the addition of Ga, hence slower cross-linking after the attack of the PAA on the glass structure. Once the disruption for the glass network occurs (due to the acid attack), Zn^2+^ and Ca^2+^ ions begin releasing before complete cross-linking occurs to set the cement. This was shown by FTIR to occur up to 30 days, hence ingress of the Ca^2+^ and Zn^2+^ ions into the distilled water can be most easily achieved with the addition of 0.16 mol% Ga. Figure 3c shows the Si release from the cements. The Si release was found to increase with maturation. Similar results were obtained for the Control and LGa-1 cements over all periods; however a relatively high release of Si was obtained for the LGa-2 cements over 1, 7 and 30 days, peaking at ~28 ppm. As discussed earlier the higher concentration of Si release for the LGa-2 is caused by the longer setting reaction and easier solubility of these ions during the setting reaction. FTIR results proved that the cements have the Si–O–Si bond. The silica bond will be formed during the setting reaction and will be loosely bound at the surface of the glass particles. These silica molecules will then dissolve when immersing the cement samples in the distilled water, hence Si^4+^ ions will ingress into the water relatively easily. This process was found to be fast for the first 7 days for the LGa-2 cement, hence facilitating the relatively high concentration obtained for the Si release from LGa-2 cement. While it should be considered that the Si release did not increase significantly for 30 days evaluation for LGa-2 cement. Figure 3d shows the Ga^3+^ release from the cements. No Ga^3+^ release was obtained in the Control cement as expected, however it was found that there was no Ga^3+^ release for LGa-1 over 1 day evaluation. Additionally, very low concentrations of Ga^3+^ release was detected for 7 days (~0.2 ppm) and 30 days (~0.3 ppm) evaluation of LGa-1. Ga^3+^ release from LGa-2 cements presented low concentration (~0.4 ppm) of Ga^3+^ ions after 1 day, but unlike LGa-1, LGa-2, presented relatively high concentrations of Ga^3+^ after 7 days (~3 ppm) and 30 days (~3 ppm). The low concentration of Ga^3+^ release, when compared to other ions, may be due to the low maturation behavior of the Ga^3+^ ions within the cements. The addition of Ga causes an incomplete cross linking of the cement upon initial setting due to the slower reaction of the Ga^3+^ ions with the carboxylic groups. The short mixing time (~30 s) of each of these cements seem to allow the Zn^2+^ and Ca^2+^ (divalent cross linking) ions to be released adequately, however this might be limiting the release and mobility of the Ga^3+^ (trivalent cross linking) ions. One affect this may have is the mobility of Ga ions. Trivalent ions will have a lower mobility than the Ca^2+^ and Zn^2+^ ions and therefore will not reach or completely crosslink during the short mixing and fast setting (~250 s) times. 

Overall, the low release profile of Ga^3+^ ions (~3 ppm) is sufficient to bind Ga^3+^ to the DNA phosphate, forming a stable complex [66]. This is important in achieving better clinical outcomes that will reduce the post-operative complications as sufficient release of Ga^3+^ ions (~3 ppm) may inhibit the DNA synthesis by modifying its three dimensional (3D) structure, the modulate protein synthesis, and the activity of some enzymes such as DNA polymerases, ATPases, tyrosine-specific protein phosphatise and ribonucleotide reductase [41]. On the other hand, this low concentration of ion release is necessary to avoid the reported toxicity of Ga ions [67].

**Figure 4:**
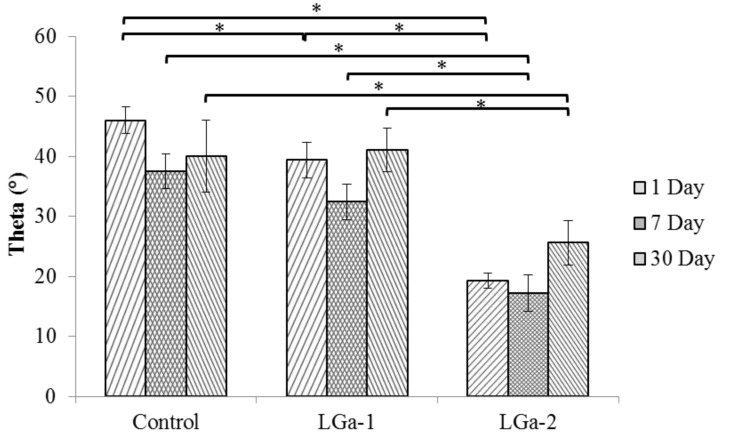
Contact angle measurements for the cement series over 1, 7 and 30 days. Stars and bars show statistical significance (p < 0.05).

Contact angle measurements were recorded to determine the tendency of the cement surface to absorb water (hydophilicity) in relation to Ga incorporation in the glass precursors, hence evaluating the cement surface chemistry. Figure 4 shows the contact angle measurements of the cement series over 1, 7 and 30 days. The contact angle was found to decrease over 1, 7 and 30 days. It can also be seen that the addition of Ga in the glass composition appears to reduce the contact angle of the cement. The Control glass was found to have the highest contact angle over 1 (~46°), 7 (~37°) and 30 (~40°) days in comparison to LGa-1 and LGa-2; however, there was no statistically significant difference between Control and LGa-1 for 7 days (Р = 0.057) and 30 days (Р = 1.000). Hence, confirming the results and discussions regarding the quicker set and maturation of the Control glasses when compared to LGa-1 and LGa-2. The quicker setting reaction of the Control glasses results in quicker bond formation between the glass network and the carboxylic groups. As a consequence, this affects the ability of the cement structure to react with the surrounding water molecules reduces. According to Mittal [68], the wettability of the surface increases with the decrease in contact angle. Contact angles <90° are considered hydrophilic. Further, lower contact angles are associated with both higher roughness and adhesion-ability. In general, all cement samples are hydrophilic. However, hydrophilicity increases with Ga concentration in the glass series. 

**Figure 5:**
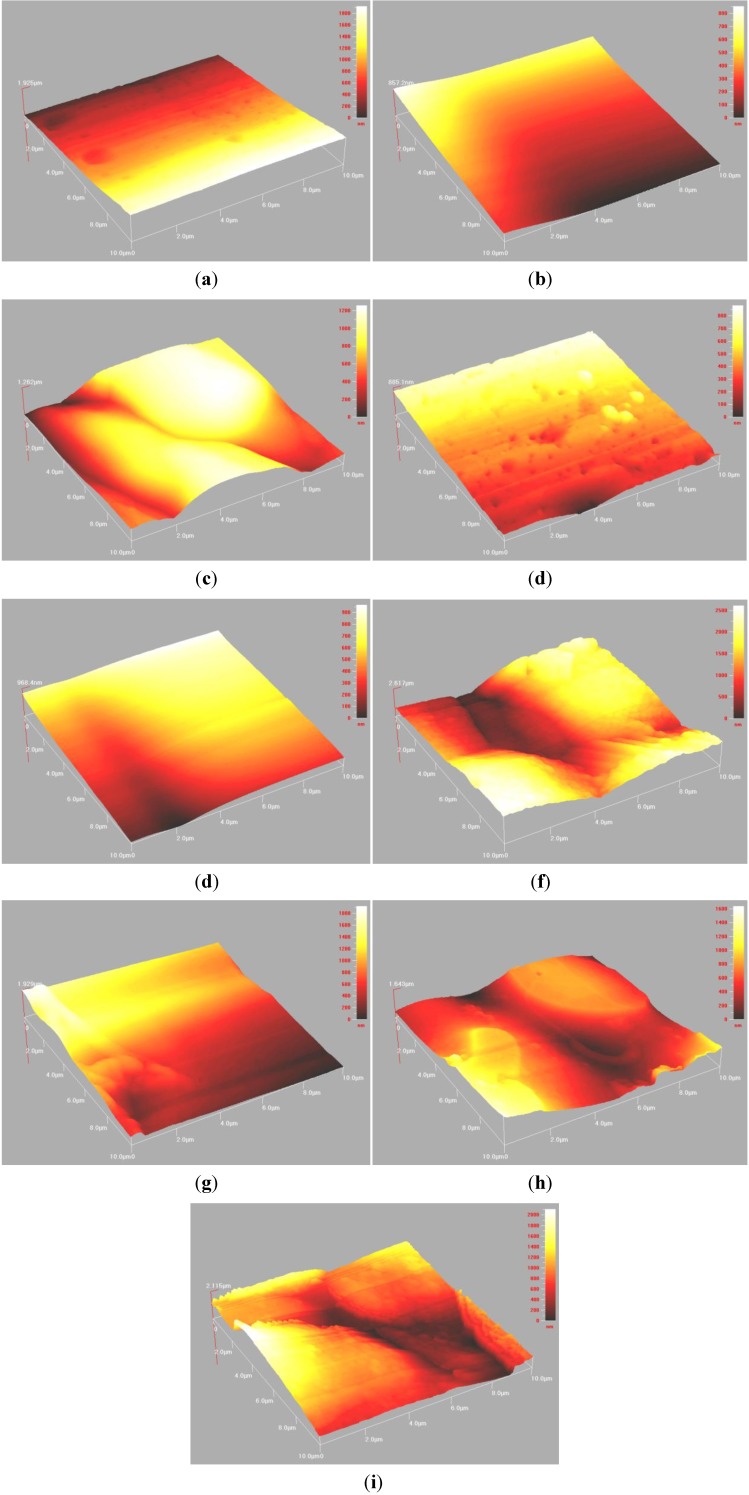
3D surface morphology of the cement series observing (**a**) Control; (**b**) LGa-1; (**c**) LGa-2 for 1 day evaluation; (**d**) Control; (**e**) LGa-1; (**f**) LGa-2 for 7 day evaluation; (**g**) Control; (**h**) LGa-1; and (**i**) LGa-2 for 30 day evaluation.

**Figure 6:**
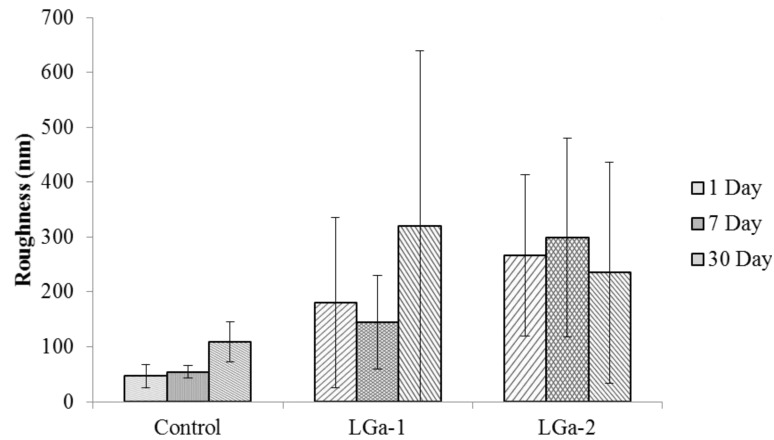
Roughness measurements for the cement series over 1, 7 and 30 days. Stars and bars show statistical significance (p < 0.05).

Atomic force microscopy (AFM) was important for evaluating surface morphology and roughness (Ra). Figure 5 shows the 3D surface morphology of the cement series over 1, 7 and 30 days. The coloured section refers to the magnitude of the Roughness (Ra) in nm. Figure 6 shows the Ra of the cement series over 1, 7 and 30 days. Figure 5a–c show the 1 day surface morphology. It can be seen that the Control cement (Figure 5a) is more granular in comparison to LGa-1 and LGa-2 represented by different size holes distributed on the surface. It can also be seen that the step height is dissimilar throughout the surface and hence representing a higher Ra at one end. Figure 5b,c display more homogeneous surfaces in comparison to the Control cement. Similar results with higher Ra can be realized in Figure 5d–f for the 7 days surface evaluation. However the surface morphology of the LGa-2 presented some elevations at points of the surface. This might be due to the longer maturation time of the cement. Figure 5g-i show the 30 days surface morphology. Similar to 1 and 7 days evaluation, results for 30 days show that the Control cement is more homogeneous when compared with 1 and 7 days evaluation. It can also be seen that the surface morphology for LGa-1 and LGa-2 are similar with low Ra and high Ra points. Interestingly, it was seen that the addition of Ga to the cement series increases Ra according to the values obtained from Figure 6. Ra values are ~47, 180 and 266 nm for Control, LGa-1 and LGa-2 respectively for 1 day measurements. For 7 day measurements the Ra increased from ~54 nm for Control to ~145 nm for LGa-1 and to ~299 nm for LGa-2. Measurements for 30 days presented similar results, where the Ra data are ~109, 321, 235 nm for Control, LGa-1 and LGa-2 respectively. It can be seen from Figure 6 that there was no statistical significant difference between the Ra of the Control, LGa-1 and LGa-2 cements, respectively. AFM results indicate that the increased setting reaction due to the addition of Ga results in increased Ra. This may be due to the network disruption occurring during the increased maturation time of the Ga samples. The behaviour of Ga is expected to have a similar structural role to that of the Al such that the Ga^3+^ ions partially replace the Si^4+^ ions, imparting a negative charge on the glass network. Since Ga can act as a network modifier, it is expected that Ga^3+^ disrupts the connectivity of Si–O–Si network by introducing non-bridging oxygen species (NBOs) [24], as shown in the FTIR results. These results confirm the results of the contact angle measurements and prove the discussions presented by Mittal [68].

**Figure 7:**
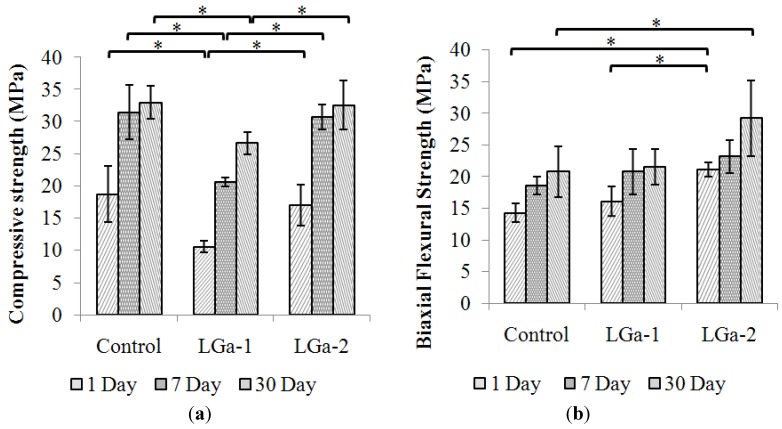
Mechanical properties (**a**) compressive; and (**b**) biaxial flexural strength survey results for the cement series over 1, 7 and 30 days. Stars and bars show statistical significance (p < 0.05).

Figure 7 presents the mechanical properties of the cement series over 1, 7 and 30 days. Figure 7a shows the compressive strength (σ_c_) while Figure 7b shows the biaxial flexural strength (σ_f_) results. In general, similar to the results presented in the literature [24], the Control glass has the highest strength, LGa-1 has the lowest and LGa-2 has higher strength than LGa-1 and lower than, but comparable to, the cement based on the Control glasses. According to FTIR, all the samples have similar levels of water content at 1 day, and identical water contents when aged 7 and 30 days. Thus the change in the mechanical properties does not seem to be dependent on the water content. It is highly likely that the Control samples experience greater strengths due to the increased levels of cross-linking when compared to the LGa 1 and 2 samples, however this does not explain the increased σ_c_ experienced by the LGa-2 samples compared to the LGa-1 samples. Both Ga containing sample sets experience very similar FTIR traces with similar levels of cross-linking taking place across all aged samples. Thus the reason for the increase in σ_c_ of the LGa-2 samples compared to the LGa-1 samples is unknown in this study and would need further investigation. 

σ_f_ obtained display slight improvements between Control and LGa-2 samples over the 1, 7 and 30 day samples. In accordance with the results presented by Wren *et al.* [24], the addition of Ga was found to decrease both σ_c_ and σ_f_. However there was no statistical significant difference between Control and LGa-2 for 1, 7 and 30 days measurements of σ_c_ and for 7 days measurement of σ_f_.

**Figure 8:**
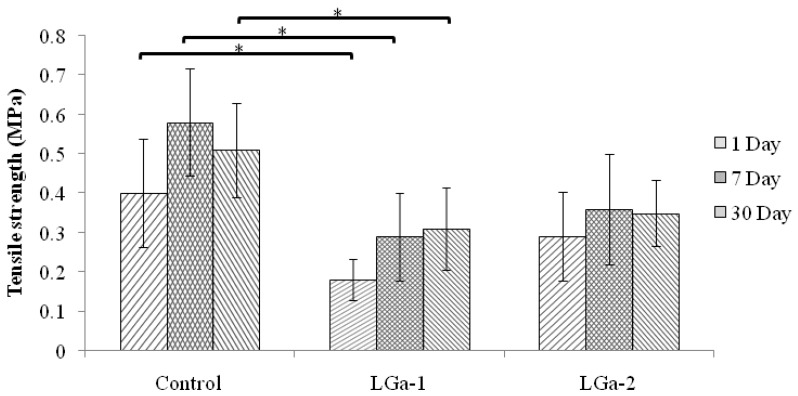
*Ex-vivo* tensile strength measurements for the tensile tested bones over 1, 7 and 30 days. Stars and bars show statistical significance (p < 0.05).

The work seeks the development of therapeutic Ga-containing GPCs for fixation purposes in the skeleton, particularly in the incidence of sternal closure. Figure 8 shows the *ex-vivo* tensile strength measurements for all cement series over 1, 7 and 30 days. As can be seen, the strength was found to increase after 1 day; however, 30 day measurements show similar results to the 7 day measurements. Additionally, it can be observed that the strength for LGa-1 decreases significantly while it increases for LGa-2. The strengths for Control cements were ~0.4, 0.6 and 0.5 MPa for 1, 7 and 30 days measurements, respectively. For LGa-1, the strengths are ~0.2, 0.3 and 0.3 MPa while LGa-2 strength values are ~0.3, 0.4, 0.4 MPa respectively, over the same periods. There is a statistical significant difference between Control and LGa-1, but there was no significant difference between Control and LGa-2. The increase in strength for LGa-2 may be related to the longer setting reaction that facilitates improved cross-linking and due to the higher mol% of the Ga, which is expected to have a similar behaviour to Al^3+^ ions in the GPCs due to the same valence state and hence increasing the strength. This indicates that LGa-2 may be used instead of the Control glass due to the comparable strength values, however its use as an alternative to Kryptonite is still questionable and would require further work. It was reported [69] that the indirect measurements for the forces imposed on the sternum resulted in a force of 260 N during a ~5.6 kPa pressure generating cough. It was also presented that forces from 160 N to 400 N and 550 N to 1650 N are imposed on the sternal midline during breathing and coughing, respectively. Kryptonite cement, supported by wires, was able to resist ~600 N [4], whereas wires alone showed ~2 mm displacement at ~400 N. Our Ga-GPCs resulted in lower strength values, considering LGa-2, that are ~100, 69 and 92 N for 1, 7 and 30 days assessment respectively. However, our study did not consider the use of wires. Ideally a study directly comparing the strength of the LGa-2 samples to Kryptonite would be needed.

**Figure 9:**
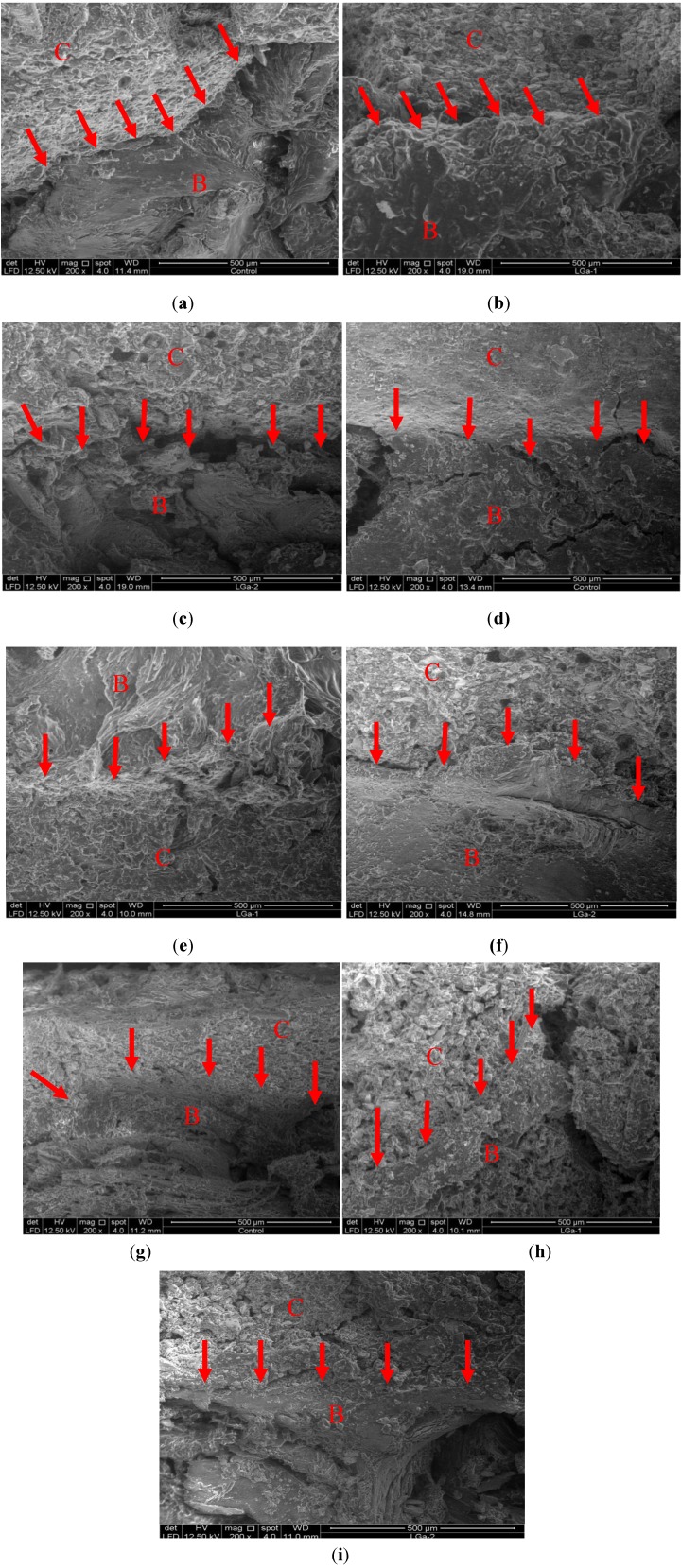
SEM cross-sectional images for the tensile tested bones, observing (**a**) Control; (**b**) LGa-1; (**c**) LGa-2 for 1 day evaluation; (**d**) Control; (**e**) LGa-1; (**f**) LGa-2 for 7 days evaluation; (**g**) Control; (**h**) LGa-1; (**i**) LGa-2 for 30 day evaluation. The red arrows point towards the attachment between the cement and the bone marrow while the letters C and B refer to cement and bone, respectively.

Further investigations also included evaluating failure mechanism using the scanning electron microscopy (SEM). Energy dispersive x-ray (EDX) analysis was also performed to obtain the wt% of each of the ions incorporated at the starting glass. Figure 9 shows the SEM images for the cement series over 1, 7 and 30 days. In Figure 9 the red arrows point toward the attachment between the cement and the bone marrow while the letters C and B indicate the area of cement and bone, respectively. The cement area can be clearly seen and differentiated from that of the bone by its granular and brighter view. Figure 9a–c show the SEM images for the attachment between the bone and Control, LGa-1 and LGa-2 cements respectively over day 1. Figure 9d–f show the SEM images for the attachment between the bone and Control, LGa-1 and LGa-2 cements respectively over 7 days. Figure 9g–i show the SEM images for the attachment between the bone and the Control, LGa-1 and LGa-2 cements respectively over 30 days. It can be observed that the failure is not due to adhesive strength as the cement is completely attached to the bone in all images. It can be also seen that the attachment improves with cement maturation. However, it must be considered that the integration is not similar to that of the normal living bone. In general it can be seen that the Ga-containing cements bond completely to the bone observed at all periods, while the 7 and 30 day images show better attachment when compared to day 1.

Table 1 shows the wt % of the ions over 1, 7 and 30 days observed from the EDX survey measurement. The Ca^2+^ concentration values are ~17%, 10% and 11% for Control, LGa-1 and LGa-2 respectively over day 1, ~22%, 20% and 17% over 7 days and ~32, 19 and 17% over 30 days. It can be seen that the concentration increases with maturation for all cements except for LGa-1 and LGa-2 at 30 days. The increase in the concentration with cement maturation was expected due to the relevant results of the ion release. However it was not expected that the Ca^2+^ concentration decreases with the addition of Ga. The ion release profiles of Ca^2+^ displayed increased concentration of Ca^2+^ ions in water with the addition of Ga, particularly with LGa-2. Relating this to the *ex-vivo* study, this might be caused by the adsorption of the Ca^2+^ ions by the bone during maturation. The Zn^2+^ concentration values are ~55%, 39% and 23% for Control, LGa-1 and LGa-2 respectively over day 1, ~46%, 37% and 24% over 7 days and ~48, 38 and 25% over 30 days. It can be seen that the concentration values of Zn^2+^ are of a similar trend to those presented for the Ca^2+^ over all periods. Further, the addition of Ga was found to decrease the wt% of Zn^2+^ ions. It can also be seen that the concentration of Zn^2+^ over all periods for all cements is higher than that of the Ca^2+^. The Si concentration values were found as ~27%, 26% and 37% for , LGa-1 and LGa-2 respectively over day 1, ~32%, 25% and 26% over 7 days and ~36%, 25% and 23% over 30 days. The Si concentration increased over all periods for the Control glass, remained similar for LGa-1 and decreased over all periods for LGa-2. The results of the Si concentration did not follow the same trend as those of the Ca^2+^ and Zn^2+^. The Ga^3+^ concentration values were according to the authors’ expectations. The Control cement has zero wt% while for the LGa-1 and LGa-2 the concentration of Ga^3+^ increased with the addition of Ga. Ga concentration values were ~0, 25 and 28% for Control, LGa-1 and LGa-2 respectively over 1 day, ~0%, 18%, and 39% over 7 days and 0%, 18% and 36% over 30 days. Results of Ga concentration are in line with the results achieved from the ion release study. However, the high concentration of LGa-1 (25%) over 1 day was not expected since there was no ion release from LGa-1 after 1 day. In general, the EDX of the tested bone samples provided an indication of the presence of all incorporated ions over all periods. 

**Table 1:** Energy dispersive x-ray (EDX) survey results observing the wt % for the Ca^2+^, Zn^2+^, Si^4+^ and Ga^3+^ ions over 1, 7 and 30 days.

Ion Species	Control			LGa-1			LGa-2		
	1	7	30	1	7	30	1	7	30
Ca^2+^ (wt %)	17.1	22.4	32	10.4	19.6	19.2	11.1	17.3	16.7
Zn^2+^ (wt %)	55.5	45.6	48.2	38.9	37.2	37.7	23.0	23.8	24.5
Si^4+^ (wt %)	27.4	32.0	36.1	25.7	25.1	24.8	37.4	25.8	22.9
Ga^3+^ (wt %)	0.0	0.0	0.0	25.1	18.2	18.3	28.4	39.3	35.9

## 3. Experimental Section

### 3.1. Glass Synthesis

Three Ga containing glass compositions (Control, LGa-1, and LGa-2) were formulated. The Control was a Ga-free CaO-ZnO-SiO_2_ glass, LGa-1 and LGa-2 contain incremental concentrations of Ga added into this Control composition at the expense of Zn (Table 2). Glasses were prepared by weighing out appropriate amounts of analytical grade reagents and ball milling (1 h). The mix was then oven dried (100 °C, 1 h) and fired (1500 °C, 1 h) in a platinum crucible and shock quenched into water. The resulting frit was dried, ground and sieved to retrieve a glass powder with a maximum particle size of <45 μm.

**Table 2:** Glass compositions (mol%).

Composition	Control	LGa-1	LGa-2
SiO_2_	0.48	0.48	0.48
Ga_2_O_3_	0.00	0.08	0.16
ZnO	0.40	0.32	0.24
CaO	0.12	0.12	0.12

### 3.2. Glass Characterization

#### 3.2.1. X-Ray Diffraction

Diffraction patterns were collected using a Siemens D5000 X-ray diffractometer (Bruker AXS Inc., Fitchburg, WI, USA). Glass powder samples were packed into standard stainless steel sample holders. A generator voltage of 40 kV and a tube current of 30 mA were employed. Diffractograms were collected in the range 10° < 2θ < 70°, at a scan step size 0.02° and a step time of 10 s.

#### 3.2.2. Particle Size Analysis

PSA was achieved using a Beckman Coulter Multisizer 4 Particle size analyzer (Beckman Coulter Inc., Fullerton, CA, USA). Glass powder samples were evaluated in the range of 0.4–100 μm with a run length of 60 s. The fluid used was distilled water at a temperature range between 10–37 °C. The relevant volume statistics were calculated concerning each glass.

### 3.3. Cement Preparation

Cement samples were prepared by thoroughly mixing the glass powder (Section 3.1) with E11 PAA (PAA-Mw, ~120,000 and <90 μm, Sigma-Aldrich, St. Louis, MI, USA) and distilled water on a glass plate. The cements were formulated in a powder: liquid (P:L) ratio of 1:0.74 with 50 wt % additions of PAA; considering that the powder is the glass series and the liquid is the PAA and water mixture, where 1 g of glass powder was mixed with 0.37 g E11 PAA and 0.37 ml water. Complete mixing was undertaken within 30 s in ambient room temperature (23 ± 1 °C). The cements are now reported with the same nomenclature (Control, LGa-1, and LGa-2) that was assigned to the glasses that they were fabricated from.

### 3.4. Rheological Properties

#### 3.4.1. Net Setting Time

The T_s_ of 3 samples for each cement formulation were tested in ambient air (23 ± 1 °C) according to ISO 9917-1:2007. Briefly, the net setting time was defined as the time from the end of mixing and until the material is completely set [70]. 

#### 3.4.2. Working Time

The T_w_ of 3 samples for each cement formulation were measured in ambient air (23 ± 1 °C) using a stop watch according to the method described by [71]. Briefly, the working time was defined as the time from the start of mixing, through which the material can be manipulated without having an adverse effect on its properties [70].

### 3.5. Cement Characterization

#### 3.5.1. Fourier Transform Infrared Spectroscopy

Three cement cylinders (6 mm high and 4 mm diameter) were prepared from each glass type and aged for 1, 7 and 30 days in distilled water (no specific volume since the volume does not affect the properties of these materials) before FTIR analysis. The analysis required a powder with a mean particle size <90 μm. Hence, a pestle and mortar were used to grind the cement. The spectra were collected 5 times for each cement formulation in ambient air (23 ± 1 °C).

The spectra were collected using an FTIR spectrophotometer-Thermo Scientific-Nicolet iS10 (Thermo Fisher Scientific, Waltham, MA, USA) equipped with a room temperature Deuterated Tri-Glycine Sulfate (DTGS) KBr detector. Analysis was performed in the wave-number ranging from 650 to 4000 cm^−1^ with a spectral resolution of 4 cm^−1^. 

#### 3.5.2. Ion Release Studies

Ion release studies were performed according to a method described by Wren *et al*. [23]. The ion release profiles of GPC samples (3 samples for each cement in the series) were measured using the Agilent 4100 (Agilent Technologies, Inc., Santa Clara, CA, USA) microwave plasma–atomic emission spectrometer (MP–AES). MP–AES calibration standards for Ga^3+^, Ca^2+^, Zn^2+^ and Si^4+^ elements were prepared from a stock solution on a gravimetric basis. Three target calibration standards were prepared for each ion with 0.3, 0.5 and 1.0 ppm concentrations while distilled water was used as a blank. Samples for Ca^2+^, Zn^2+^ and Ga^3+^ analysis were diluted in a ratio of 1:10; that is, each 1 ml of concentrated sample was mixed with 10 ml of distilled water while samples for Si analysis were diluted in a ratio of 1:30. A pilot study was conducted to determine the appropriate ratio for dilution of all elements. 

#### 3.5.3. Contact Angle Measurements

GPC discs (1 mm high and 12 mm diameter, where n = 5) were prepared from each glass type for the contact angle measurements. Contact angles were measured by a method described by Moshaverinia *et al*. [72]. Disc samples were tested after being aged for 1, 7 and 30 days in distilled water in a 37 °C incubator. Measurements were taken using an OCA 20 optical contact angle measuring instrument (DataPhysics Instruments GmbH, Filderstadt, Germany). The distilled water drops were deposited with a micro-syringe (DataPhysics Instruments GmbH, Filderstadt, Germany) on each sample.

#### 3.5.4. Atomic Force Microscopy

A single GPC disc (1 mm high and 15 mm diameter) was prepared from each glass composition for the AFM survey analysis. Measurements were taken at 3 different sites on each sample. Samples were tested after being aged for 1, 7 and 30 days in distilled water in a 37 °C incubator. AFM was performed using Ambios Q-Scope^TM^ 250/400 Nomad^TM^ Series Atomic Force Microscope (Ambios Technology Inc., Santa Cruz, CA, USA). Intermittent-contact (wave) mode imaging was performed using a silicon nitride cantilever probe. A typical scan rate of 1 Hz and a scan size of 10 μm were used at a resolution of 256–512 pixels/line.

### 3.6. Mechanical Properties

#### 3.6.1. Compressive Strength

σ_c_ of five GPC samples were evaluated in ambient air (23 ± 1 °C) according to ISO 9917 [70]. Cylindrical samples were tested after being aged for 1, 7 and 30 days in distilled water in a 37 °C incubator. Testing was undertaken on an Instron Universal Testing Machine (Instron Microtester 5848, Instron Corp., Norwood, MA, USA) using a ±2 kN load cell at a crosshead speed of 1 mm∙min^−1^. 

#### 3.6.2. Flexural Strength

The σ_f_ of 5 GPC samples were evaluated in ambient air (23 ± 1 °C) by a method described by Williams *et al*. [73]. 1 mm thick discs were made in Poly(methyl methacrylate) (PMMA) moulds, measuring 22.5 mm diameter. Each mould was filled with cement within 1 min of mixing, compressed between PMMA plates and left clamped at 37 °C and > 80% relative humidity. Not later than 60 min after the end of mixing, the cement samples were removed from the mould and placed in distilled water at 37 °C for a further 23 h before testing.

**Figure 10:**
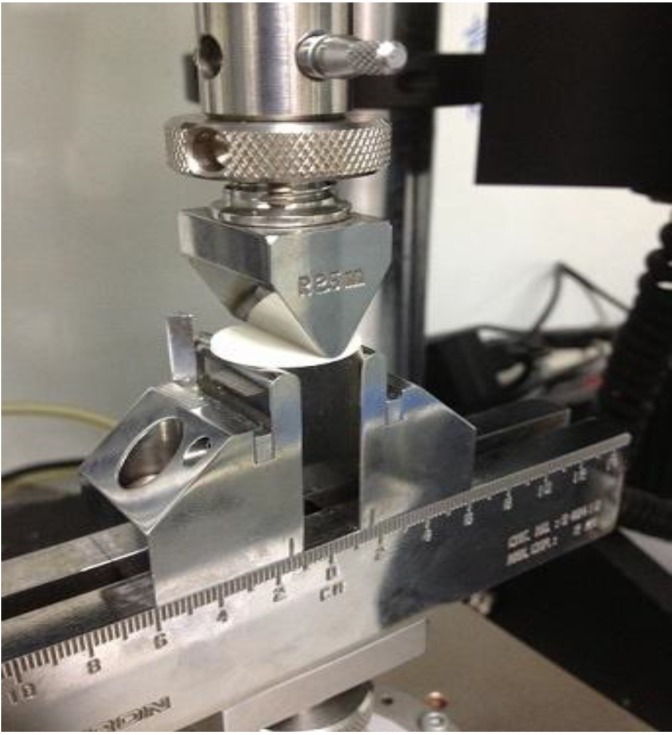
The cross head indenter used for biaxial flexural test.

Disc samples were tested after 1, 7 and 30 days. Testing was undertaken on an Instron Universal Testing Machine (Instron Microtester 5848, Instron Corp., Norwood, MA, USA) using a ±2 kN load cell at a crosshead speed of 1 mm∙min^−1^. The crosshead indenter is a knife edge of 2.5 mm diameter (Figure 10). The fixture type is a 3-point support allowing the load to be applied at the center of the disc. The discs are positioned equidistant around the 15 mm support span diameter, with only 3 mm of unsupported cement on each side of the support span.

### 3.7. *Ex-vivo* Study

#### 3.7.1. Sample Collection and Preparation

**Figure 11:**
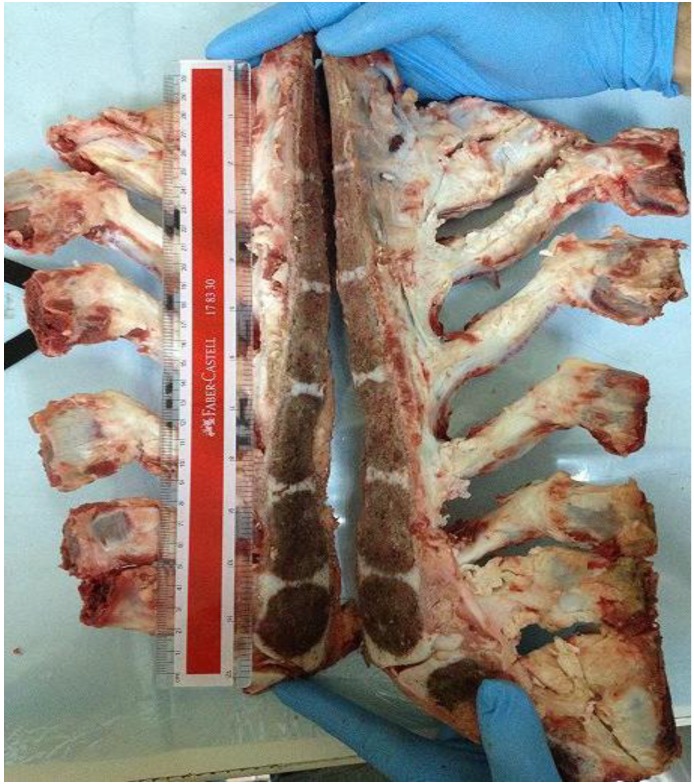
The bovine sternal model.

This study utilized a biological bovine sterna model (Figure 11). Sterna were harvested from freshly slaughtered bovines (Slaughterhouse and Meat Products, Shah Alam, Malaysia). All animals were in normal condition with ages ranging between 2 to 3 years. A midline incision in the sternum was made using an oscillating power saw (EFA Meat Processing Power, Maulbronn, Germany). The sternum halves were then collected and stored at −80 °C. 

**Figure 12:**
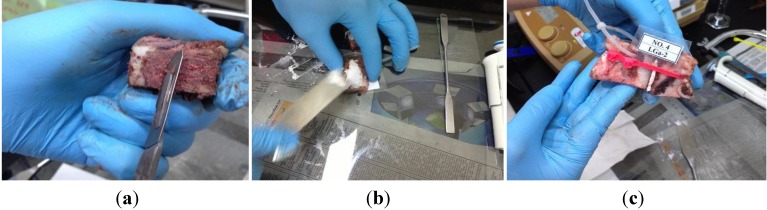
Sample preparation steps. (**a**) Surface treatment; (**b**) Cement application; and (**c**) Approximation, labelling and clamping.

Prior to specimen preparation, sterna were stored at 0–5 °C for 24 h. The specimens were prepared by detaching the ribs and the surrounding tissues using a universal band saw machine (L-300, Luxo Corp., Elmsford, NY, USA). Each sternum was utilised to obtain 5–6 specimens by cutting the sternal halves horizontally. Figure 12 shows the surface treatment and sample preparation steps. A surgical blade (Ribbel International Ltd., Kuala Lumpur, Malaysia) was used to remove any tissue remaining on the surface of the bone marrow (Figure 12a).The cement was then prepared and applied on the sternal halves using a spatula (Figure 12b). Labelled symmetrical halves were than approximated together, and clamped using cable ties (Butterworth Eng Tat Trading Sdn. Bhd., Kuala Lumpur, Malaysia) (Figure 12c).

Five samples were prepared for each GPC type. Samples were then submersed in a single container of phosphate buffered saline (PBS) dissolved in distilled water according to company instructions (Sigma-Aldrich, KL, Malaysia) with 1% formaldehyde (Mw~30, 37 wt% sol. in water, stab. with 10%–15% methanol, Acros Organics, KL, Malaysia). PBS was used to mimic the physiological fluid because it is associated with fewer deleterious effects when compared with the distilled water [74], while the formaldehyde was used to prevent the growth of bacteria or fungus [75,76]. The container was kept at 37 °C. The same procedure was followed for 1, 7 and 30 days’ investigations.

#### 3.7.2. Tensile Failure Test

**Figure 13:**
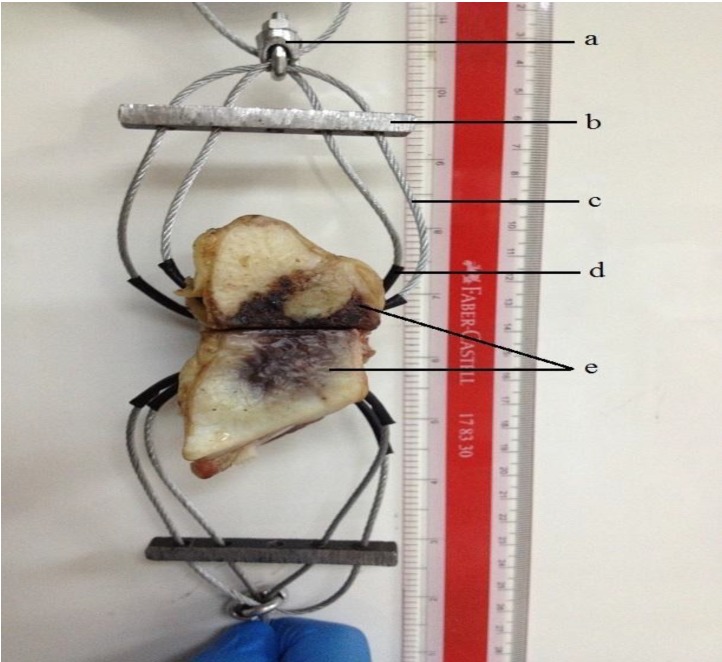
The design model for tensile failure test. (**a**) A nut to fix all wires together; (**b**) metal rod to hold all wires and distribute tension force; (**c**) bicycle’s brake cable; (**d**) a sheath to reduce the incidence of cable break through the bone; and (**e**) sternal halves.

A model was designed (Figure 13) for tensile failure test of the bovine sterna, where two correspondent holes were drilled into each side of the sternal halves to facilitate the insertion of brake cables. 

The tensile testing of 5 samples was performed in ambient air (23 ± 1 °C). Samples were tested after 1, 7 and 30 days. Testing was undertaken on an Instron Universal Testing Machine (Instron Microtester 5848, Instron Corp., Norwood, MA, USA) using a ±2 kN load cell at a crosshead speed of 1 mm∙min^−1^. The nuts (Figure 13a) were directly gripped from the Instron jaws. The tensile strength was calculated using Equation 1:

Tensile strength (MPa) = Load at break/(original width × original thickness)
(1)

#### 3.7.3. Scanning Electron Microscopy and Energy Dispersive x-ray Analysis

Samples tested for the tensile strength were stored at −80 °C for 1 h prior to freeze-drying them using the freeze dryer (Lyph-Lock 6L, Labconco Corp., Kansas City, MO, USA) for 24–48 h. Prior to the SEM-EDX analysis, a universal band saw machine (L-300, Luxo Corp., Elmsford, NY, USA) was used to prepare the samples for the SEM. Samples were cut vertically so that the adhesion between the cement and bone is seen from the internal side (a cross-sectional view). Samples from the freeze dryer were tested directly for the EDX with no other preparation techniques. 

SEM-EDX analysis was undertaken with an FEI Co. Quanta 200F Environmental Scanning Electron Microscope (Oxford Instruments X-max, Eindhoven, Netherlands). SEM images were collected at 12.5 kV. All EDX spectra were collected at 20 kV using a beam current of 26 nA. Quantitative EDX spectra were subsequently converted into relative concentration data.

### 3.8. Statistical Analysis

One way analysis of variance (ANOVA) was used to analyze the data. *Post-hoc* Bonferroni test was used to compare the relative means and to report the statistically significant differences when p < 0.05. Statistical analysis was performed using SPSS software (IBM SPSS statistics 21, IBM Corp., Armonk, NY, USA).

## 4. Limitations of the Study

- During the FTIR experiment, it was not possible to identify a band below 650 cm^−1^ due to the limited wavelength band of the instrument ranging between 650 and 4000 cm^−1^. Further research is required since Ga^3+^ particles might absorb the IR at lower wavelengths around 450 cm^−1^ and there is no research into the IR absorption for the stretching vibration between Ga^3+^ and the PAA.
- Results from ion release studies formed the hypothesis that LGa-2 will contribute to a significant reduction in the post-operative complications, post sternotomy. However, further anti-bacterial and cell culture studies are required to test this hypothesis.
- Our *ex-vivo* study does not consider osteoporosis, bleeding and bone resorption; complications regularly encountered during sternotomy fixation and repair.
- The tensile tests were performed on small samples with an average cross-sectional-area of 350 mm^2^. However, the application of Ga-cements on the complete sternum would be expected to provide higher strength due to the bond that will be formed across the whole sternum. In the same context, due to the small sample size, authors performed several pilot studies to find the best way to perform the tensile failure test. The Authors found it difficult to test the samples by attaching the sternal ribs to the Instron jaws and hence this is considered as one of the main reasons for the low strengths achieved. The forces imposed by the sternal ribs distribute across the area while in our case, the force is exerted on a small cross-sectional area of the sternum.

## 5. Conclusions

The role of Ga in the glass phase of a series of GPCs was investigated and an *ex-vivo* study utilizing bovine sterna was performed. LGa-2 has characteristics most appropriate for sternotomy applications out of the tested cements. Of the three cement compositions, LGa-2 cements were found to facilitate the greater ion release of Ca^2+^, Zn^2+^, Si^4+^ and Ga^3+^ ions. The increased release profiles of these known therapeutic ions with Ga addition may contribute to increased healing due to the anti-bacterial and anti-inflammatory nature of Ga^3+,^ and the additional anti-bacterial nature of Zn^2+^. Furthermore, the longer handling properties of LGa-2 were found to affect the material positively, giving the surgeon sufficient time to apply the cement, while the kinetic reactions are not affected. The mechanical strength of the LGa-2 samples displayed improved biaxial flexural strength, and comparable compressive strength, to the Control glasses. However these strengths are too low to use this glass without the use of wires for sternal fixation. Further research would be necessary to test the suitability of this glass in conjunction with wire closure. Nevertheless, this initial study has shown that Ga based glass may have the potential to be used for sternal fixation, in conjunction with wires in much the same manner as Kryptonite, while adding a therapeutic effect. A study directly comparing the strength to Kryptonite would also be needed to comment on the mechanical performance of the LGa glasses to a known adhesive product that is currently used in this procedure. This may be the subject of future work.
